# The Art of Surgery: Balancing Compassionate With Virtual Care

**DOI:** 10.2196/22417

**Published:** 2020-08-27

**Authors:** Elisheva Tamar Anne Nemetz, David Robert Urbach, Karen Michelle Devon

**Affiliations:** 1 Faculty of Medicine University of Toronto Toronto, ON Canada; 2 Department of Surgery Faculty of Medicine, University of Toronto Toronto, ON Canada; 3 Institute of Health Policy, Management and Evaluation University of Toronto Toronto, ON Canada; 4 University Health Network Toronto, ON Canada; 5 Women's College Hospital Toronto, ON Canada; 6 Joint Centre for Bioethics, Dalla Lana School of Public Health University of Toronto Toronto, ON Canada

**Keywords:** bioethics, medical ethics, virtual care, telehealth, virtual care in surgery, video care in surgery, telehealth in surgery, surgical communication, COVID-19 and virtual care, consent, privacy, medical education, surgery

## Abstract

The recent drive to include virtual care in surgical practice has been accelerated due to the COVID-19 pandemic. Many physicians feel that communicating via telehealth is unlike traditional methods of providing health care, and thus guidance on maintaining excellence in communication is necessary, especially as academic literature on virtual care in surgery is nonexistent. Challenges faced in transitioning to virtual care include the inability to utilize body language, barriers to traditional physical examination, exacerbation of existing vulnerabilities and inequities in patient groups, the declining quality of medical education, and the fragmentation of the multidisciplinary health care team. This paper seeks to resolve these challenges by focusing on the pillars of good communication, including preparation, professionalism, empathy, respect, and the virtual physical examination.

## Introduction

There has been a desire in the past few years to include virtual care in surgical practice to enhance communication and improve patient access to care. With the emergence of the COVID-19 pandemic, this transition has been accelerated and brings with it several challenges, including significantly different methods of communication and connection. Effective communication has always been a pillar of surgical practice and the surgeon-patient relationship. Excellent communication fosters a relationship based on trust and, therefore, superior medical care and outcomes [1,2]. Evidence suggests that surgeons who communicate with their patients more empathically and are active listeners also have better diagnostic acumen [3,4]. Better communicators also face fewer medical malpractice suits and, more importantly, have more satisfied patients [4]. The literature on virtual surgical care is scarce, and guidance in the form of academic articles, manuals, and lectures on effective virtual medical communication and compassionate virtual care is missing from the surgical discourse. Many physicians feel that communicating via telehealth is too unlike traditional methods of providing health care, and thus guidance on maintaining excellence in communication is necessary.

## Challenges in Telephone and Video Communication

Virtual communication inherently brings challenges. First, medical care provided over the phone does not permit the use of body language as a form of non-verbal communication. Additionally, a critical part of every medical encounter and physical examination is evaluating the patient’s general appearance, which begins the moment the patient is greeted and is not feasible over the phone.

Video-based medical care, while potentially mitigating some of these problems, also has limitations. Although video communication allows for non-verbal communication, the use of touch is nonetheless limited. A hand on a shoulder when performing an examination or simply being seated and present with a patient through a difficult diagnosis cannot be replicated in a two-dimensional medium. Body language speaks volumes, and surgeons rely on body language as an essential form of communication, even aside from the necessity of a physical exam.

Virtual care can also exacerbate existing vulnerabilities and inequities in patient groups. For example, those that have difficulty with hearing, who rely on sign language, or for whom English is a second language, face even greater barriers. While interpretation services can be coordinated, it becomes even more difficult to ensure compassionate care when words are lost in translation without a physical presence. Moreover, access to a phone or computer is a privilege often taken for granted. Telephone and video-based care assume that each patient has access to technology, thereby perpetuating a socioeconomic divide. While more individuals have internet access at home, most families own only one computer [5]. It is not uncommon for a family to rely on a single computer for children’s schoolwork, family entertainment, and parental work, making confidential and safe medical care via video difficult. High-speed internet is also difficult to access for many rural Americans, due to fewer providers, less wiring for broadband services, and limited options [6]. Slow internet can interrupt the provision of virtual care and necessitate reliance on phone consultation or booking a new appointment when technology fails. Monthly billing for cellular access may be too expensive for families, impairing access to mobile phones [7]. Knowledge of how to utilize technology is also often taken for granted. Many patients, particularly the elderly, have difficulty learning how to access and use video communication. Without appropriate education and assistive social support, they become disadvantaged through reliance on phone consultations, especially when video communication might be essential, such as with postoperative wound care or gait assessments.

Amid this new reality, the quality of medical education is suffering. Many surgeons are engaged in teaching fellows, residents, and medical students and find that incorporating the education of students into the existing demands and limitations of virtual care is especially challenging. Fellows, who are honing their subspecialty and independent practice skills, may lack the opportunity to see patients before the faculty surgeon. Residents may experience delays in transitioning from medical student to practicing physician, and many medical schools have removed students from hospitals, as they are considered non-essential to patient care.

Finally, surgeons often provide care in a team-based setting requiring communication not only between patient and surgeon but also with other members of the multidisciplinary health care team such as internal medicine physicians, social workers, and nurses. The involvement of team members in a collaborative and coordinated manner is essential for good medical and surgical care. Determinants of successful collaborative care include physical space, temporal arrangements, schedules, processes, and communication tools. Conversely, spatial distance, asynchronous schedules, and dependence on virtual communication between colleagues reduce the ability to engage in collaborative care [8]. Interprofessional education focusing on training and education that promotes collaboration becomes significantly reduced when moved to an online format. Configuring a virtual multidisciplinary health care team requires mitigation of distance limitations, enhancing authentic communication, integrating schedules, and modifying interprofessional education opportunities.

The challenges posed by virtual care should be weighed against the shortcomings of in-person appointments. Patients face monetary costs associated with transportation and hospital parking fees. Additional obstacles include taking time off from work, hiring a babysitter for children at home, and long wait times. Furthermore, postoperative appointments may pose difficulties for patients who become too immobilized following surgery to travel to in-person appointments. This problem is exacerbated for those who reside in rural communities, far from their surgeon’s practice.

**Consultation request.**

Case: Patient 1 is a 75-year-old woman referred to a thyroid surgeon by her family physician due to a large goiter, which is suspicious for thyroid cancer. She lives alone and speaks Arabic fluently. She is a retired pharmacist who trained in Morocco and immigrated to Canada in 1975. Her children and grandchildren live nearby, and she spends a lot of her time enjoying their company, cooking her grandchildren’s favorite foods, and reading.

## Principles of Good Communication

Providing compassionate virtual care includes the following principles: (1) preparation, (2) professionalism, (3) empathy, (4) respect, and (5) a virtual physical examination (refer to Textbox 1 for clinical case).

### Preparation

Before booking patients for virtual medical care, it is necessary to ensure the modality is appropriate for the patient. If a patient requires a diagnostic or therapeutic intervention or physical examination, virtual medical care should not be offered, and the surgeon should work with the patient to provide that care in compliance with current hospital practices. After ensuring a patient is appropriate for virtual care, proper education of the modality of care provided should be shared with easy-to-understand instructions. For example, if the surgeon uses phone-based care from a blocked number, ensure the patient understands that they should expect a call from “No Caller ID” or “Blocked Number.” Alternatively, for example, if a surgeon utilizes an online video-conferencing system for medical care, ensure the patient has access to the meeting ID and password and recommend the patient tests computer audio and video before the appointment. Encourage the patient to access instructional videos or guides and to log on at least five minutes prior in case issues arise and alert the patient that they may receive a call within a specific time range. Patients should be provided the option to obtain technological education regarding the virtual care system the hospital utilizes. Doing so permits the patient to receive video-based medical care when they otherwise would be unable to do so. Technology-readiness can take many forms from online resources to pretesting with a mock appointment. Surgeons should ensure that patients are aware that trainees may be involved in their phone or video-based care.

The standards applicable to the physician-patient relationship established in-person must be applied to virtual care. Consent for email communication needs to be obtained, and the patient informed that personal health information will be protected. This information will not be collected, disclosed, or utilized more than reasonably necessary and will be obtained securely and privately [9]. Care provided through virtual means does not replace the need for physical examination or an in-person visit for certain disorders or urgent problems. The patient should acknowledge the need to seek urgent care in an emergency department as necessary [10]. The surgeon makes the professional calculus that the risks of virtual care do not outweigh the potential benefits and is in the patient’s best interest (refer to Textbox 2 for preparation case resolution) [9].

**Coordinating the virtual consultation.**

After receiving the referral, the surgeon’s administrative assistant contacts Patient 1 to arrange an appointment. Because she lacks internet access, she is scheduled for a phone appointment and is given instructions to expect a call with the hospital’s name and is provided a half-hour time slot. Consent for communication is obtained, and it is explained to her that her medical information will be safeguarded, and her privacy is of utmost importance. She acknowledges the need to seek urgent care in the emergency department as necessary and that virtual care may not replace the need for physical examination or an in-person visit. She is informed that because it is a teaching hospital, medical trainees may be present on the call. Patient 1 understands and is asked if she would benefit from a hospital translator that speaks Arabic. She consents to trainee involvement and requests a translator, whose assistance is arranged.

### Professionalism

Expectations should be set before clinical encounters. Surgeons should choose a private, quiet, and uncluttered location. With phone and video care, visits are often shorter and more focused than in person [7,11]. Therefore, it is important to ensure that patients and their families can share their thoughts and feelings freely. Allow for patient autonomy within the clinical encounter by beginning with open-ended questions, such as “How can I help you?” or “Tell me what you would like to discuss today?” [4] This provides the patient with the opportunity to guide the beginning of the appointment by focusing on what they view as most important and promotes patient-centered care. Physicians should consider optimizing the hospital’s electronic medical record system to provide a summary of the clinical plan, a wrap-up note, or access to lab results and imaging, a compassionate approach common to in-person clinical care.

When engaging in sensitive or emotional conversations over video or phone, surgeons should utilize appropriate points in the conversation and acknowledge the patient’s verbal or non-verbal cues. In place of body language, practitioners are required to rely on their tone of voice to display empathy, warmth, reassurance, and presence. It is also beneficial to use summarizing and signposting, a statement that clarifies for the patient what to expect in the next part of the conversation. Signposting signals a transition from one phase of a conversation to another, especially before a shift in the sensitivity or seriousness of a topic. For example, after obtaining the patient’s past medical history and before assessing the disease’s impact on the patient’s activities of daily living, acknowledgment of previous hospitalizations or illnesses with empathy is paramount. The simultaneous utilization of a transition statement is essential in signaling a progression to a more sensitive discussion.

Many institutions have recognized the limitations of phone and video medical care for patients who have difficulty hearing, rely on sign language, or for whom English is a second language. Implementing virtual interpretation services is an essential component in the new virtual delivery of care. These services can include on-demand phone and video interpretation, prescheduled interpretation booking services via phone or online booking portals, and sign language interpretation. The transition of in-hospital interpretation services to an online or phone modality is crucial for accountability, confidentiality, and equitable patient care.

The methods surgeons choose for clinical encounters should ensure patient privacy. The physical space from which the surgeon is conducting the visit should be private with minimal potential for disruption. Computer calling that utilizes Facebook, FaceTime, or unencrypted methods is discouraged [9]. It is better to utilize technology such as Direct Inward System Access (DISA), which allows staff and physicians to make outbound calls that appear to come from the clinic’s phone number. Another option is to use personal phones with the number blocked by altering the phone’s caller ID settings [12].

The surgeon should prepare to respond appropriately if technology fails or is interrupted. The surgeon should calmly instruct the patient to switch from video to a phone-based modality. Multiple means of communication should be made available before beginning the appointment in case one is interrupted. Surgeons should also be educated on their hospital’s technology services and should seek opportunities to learn to engage the technology in their practice.

Amid the time-sensitive transition to virtual care during the beginning of the COVID-19 pandemic, providers were permitted to utilize the services of virtual communication companies that were previously unauthorized [13]. HIPAA (Health Insurance Portability and Accountability Act) waivers were provided in order to permit the use of these virtual communication technology companies [13]. However, hospitals and providers are continuously advised to weigh the benefits of virtual care with patient privacy. Providers must ensure that privacy regulations and principles are placed as top priorities during virtual care (refer to Textbox 3 for professionalism case resolution).

**Initiating the virtual encounter.**

Before calling Patient 1, the surgeon chooses to call from her office, which is quiet and private. The surgical fellow, resident, and hospital translator are added to the call before the patient joins, and the surgeon shares the goals of the appointment. Following this collaboration, the patient is then added in order to mitigate any technical difficulties. The surgeon introduces the surgical fellow, resident, and translator present at Patient 1’s appointment. The surgeon outlines the appointment to her and shares that the resident may ask some questions part way through the clinical encounter, to which she agrees. The surgeon begins with an open-ended question to gain an understanding of her perception of her illness, “Can you tell me what you know about why you’re here today?” Although the surgeon has a plan and is guiding the appointment, she lets Patient 1 complete statements without interruption. When moving from one section to another, the surgeon engages in signposting. For example, when the surgeon elicits the past medical history and wants a greater understanding of how the disease is affecting her quality of life, she says, “I appreciate you sharing your recent hospitalization and how that was difficult for you. I’m now going to ask you a couple of questions regarding how this may be impacting your everyday life and the activities you enjoy doing.” The surgeon is, therefore, able to engage in a conversation where she obtains the information she needs while simultaneously allowing the patient to understand and respond appropriately to the flow of the conversation.

### Empathy

Patients have feelings and fears surrounding medical decisions and illness. Compassionate patient-centered care requires the provider to assess what the patient’s feelings or fears are, what their ideas are of the illness or medical decisions they face, what the effect of the medical decisions or illness is on their life, and what the patient’s expectations are for the clinical encounter [14].

Screen sharing tools should be utilized to permit the patient to obtain a visual representation of what to expect. This tool can help demonstrate scar size with surgery, quell patients’ concerns regarding the cosmetic results of surgery, and to provide or a visual depiction of the procedure. The surgeon should gauge the patient’s comfort level through questions such as, “How much information would you like to know?” By this approach, the provider can avoid sharing imagery or excessive procedural details concerning the surgery when the patient is uncomfortable with that information. Acknowledge the patient’s flexibility for engaging in virtual care and consider sharing appreciation for the time they put aside for the visit (refer to Textbox 4 for empathy case resolution).

**Empathic communication.**

The surgeon relies on her tone to convey empathy and warmth. The surgeon assesses the patient’s idea of illness, her feelings towards it, and expectations of the clinical encounter. When the patient hesitates from the suggestion of a biopsy as part of the medical plan moving forward, the surgeon gently says, “I can see you may have concerns about the biopsy, can you tell me a little more about how you’re feeling?” Patient 1 shares that her brother had died from thyroid cancer. Through pausing and listening and being empathetic and nonjudgmental towards the patient’s emotional reaction, the surgeon conveys that they are a team and that she will do everything she can to ensure the patient’s comfort in this process. The surgeon also suggests having a family member present at future meetings if Patient 1 believes it would be beneficial.

### Respect

With phone and video clinical encounters, there is limited opportunity for body language and movements. When engaged in video care, use eye contact to demonstrate active listening [15]. Looking off-camera, or being distracted by a notebook, computer, or phone can indicate a lack of attentiveness. Nonverbal communication can convey empathy, support, and reassurance [2]. Even slight nodding or modifying facial expressions is enough to demonstrate active listening.

In the course of a phone or video session, allow the patient to speak and complete statements without interruption. Leave space for the patient to think and pause before answering any questions. Refrain from interrupting and redirecting the patient too quickly. Additionally, active listening involves genuinely concentrating on what the patient is saying, instead of passively “hearing” [14].

Health-related beliefs, values, and behaviors are shaped by race, ethnicity, socioeconomic status, sexual orientation, and physical and mental ability [16]. Surgeons need to be culturally competent to integrate these factors into the delivery of health care. Just as surgery progresses to virtual care, the component of cultural competency needs to also. This adjustment can include incorporating traditional healers, thereby recognizing the importance of various cultures in the medical experience to expanding hours of operation in order to make clinical settings more accessible to patients (refer to Textbox 5 for respect case resolution) [16].

**Demonstrating respect.**

The surgeon focuses on being present and not rushing through any step or question, as she infuses this distant modality with humanity. She allows for natural pauses and is not too quick to redirect the patient. When reviewing imaging, the surgeon states, “I am going to look at your imaging right now so there will be some silence” to demonstrate attentiveness and keep the patient informed. After the meeting concludes, the surgeon notes on the patient’s chart that she requires interpretation services, which would be set up easily and automatically moving forward. She notes that the patient has a preference for her daughter to be present at future meetings and includes the contact information of the daughter provided. Additionally, as some aspects of treatment will require visits to the hospital, social work will be contacted to arrange resources that would benefit the patient and ensure she receives patient-centered care.

### Virtual Physical Examination

The traditional physical examination is difficult to conduct through virtual care, and surgeons require creativity, in collaboration with the patient, to complete a physical examination. This creativity can take various forms, for example utilizing at-home blood pressure cuffs or asking patients to assess pulse strength, symmetry, regularity, and quality through guidance. Some examinations can technically be implemented over video but would be socially irresponsible to carry out. For example, assessing a postoperative inguinal hernia wound over video might expose the patient and compromise patient dignity. The medical profession is careful to put into practice rituals that demonstrate respect for patients, and many of these practices are impossible over video. These include leaving the room while the patient gowns and covering a patient’s side while examining another, amongst others. When the surgeon is unsure about the appropriateness of the physical examination being completed virtually due to the efficacy of a virtual physical examination or out of respect for the patient, arrangements should be made for the patient to obtain in-person care (refer to Textbox 6 for virtual physical exam case resolution).

**Physical examination in the virtual encounter.**

The surgeon shares that she would like to get a sense of Patient 1’s health through an examination. The surgeon communicates that the resident will ask her some questions in order to obtain more information about the changes she is experiencing and that some questions may require movement by the patient. She understands, and the resident guides her through checking her height, weight, temperature, blood pressure, and pulse rate. The resident also acknowledges the patient’s thyroid enlargement by asking if she or her family has noticed any change in her thyroid, and she responds that her daughter has commented on her neck getting bigger. In this particular case, the resident asks if she can move her neck and if she can touch her chin to her chest in order to determine if she exhibits a full range of motion. The virtual examination of Patient 1 elicited all the information required at this stage.

## Conclusion

The literature on virtual surgical care is sparse, and guidance in the form of academic articles, manuals, and lectures on compassionate virtual care is absent from surgical discourse thus far. Through focusing on pillars of good communication, including preparation, professionalism, empathy, respect, and the virtual physical examination, this paper sought to address these challenges. Communication is the foundation of compassionate virtual care, thus more research is required to ensure that the modality is mastered in surgical care.

## Abbreviations

DISA: Direct Inward System Access
HIPPA: Health Insurance Portability and Accountability Act

## References

[1] Weng H, Steed JF, Yu S, Liu Y, Hsu C, Yu T, Chen W (2011). The effect of surgeon empathy and emotional intelligence on patient satisfaction. Adv Health Sci Educ Theory Pract.

[2] Berman AC, Chutka DS (2016). Assessing effective physician-patient communication skills:. Korean J Med Educ.

[3] Ranjan P, Kumari A, Chakrawarty A (2015). How can Doctors Improve their Communication Skills?. J Clin Diagn Res.

[4] Kuehl SP (2011). Communication Tools for the Modern Doctor Bag. Physician Patient Communication Part 1: Beginning of a medical interview. J Community Hosp Intern Med Perspect.

[5] Pew Research Center 2016 Nov. The Typical American Household Contains Multiple Connected Devices.

[6] Anderson M (2018). About A Quarter of Rural Americans Say Access to High Speed Internet is a Major Problem. Pew Research Center Sep.

[7] Calton B, Abedini N, Fratkin M (2020). Telemedicine in the Time of Coronavirus. J Pain Symptom Manage.

[8] Morley L, Cashell A (2017). Collaboration in Health Care. J Med Imaging Radiat Sci.

[9] College of PhysiciansSurgeons of Ontario (2000). Protecting Personal Health Information. Policies 11-15.

[10] Virtual Care Telephone and Video Codes Frequently Asked Questions 2020 Mar. Ontario Medical Association (OMA).

[11] Levinson W, Chaumeton N (1999). Communication between surgeons and patients in routine office visits. Surgery.

[12] Rosh J, Mehta N Magenta Health Case Study - Google Docs 2020 Apr.

[13] U.S. Department of Health & Human Services 2020 Mar. Notification of Enforcement Discretion for Telehealth Remote Communications During the COVID-19 Nationwide Public Health Emergency.

[14] Grek A, Hudson J, Tzanetos K, Wong D (2019). Communication Skills 1 Initiate the Encounter. University of Toronto Faculty of Medicine. PDF.

[15] Calton BA, Rabow MW, Branagan L, Dionne-Odom JN, Parker Oliver D, Bakitas MA, Fratkin MD, Lustbader D, Jones CA, Ritchie CS (2019). Top Ten Tips Palliative Care Clinicians Should Know About Telepalliative Care. J Palliat Med.

[16] Cultural Competence in Health Care: Is it important for people with chronic conditions?.

